# Mitigating Dietary Bisphenol Exposure Through the Gut Microbiota: The Role of Next-Generation Probiotics in Bacterial Detoxification

**DOI:** 10.3390/nu16213757

**Published:** 2024-10-31

**Authors:** Paulina Emanowicz, Paulina Średnicka, Michał Wójcicki, Marek Roszko, Edyta Juszczuk-Kubiak

**Affiliations:** 1 Laboratory of Biotechnology and Molecular Engineering, Department of Microbiology, Prof. Wacław Dąbrowski Institute of Agricultural and Food Biotechnology–State Research Institute, Rakowiecka 36 Street, 02-532 Warsaw, Poland; paulina.srednicka@ibprs.pl (P.Ś.); michal.wojcicki@ibprs.pl (M.W.); edyta.juszczuk-kubiak@ibprs.pl (E.J.-K.); 2 Department of Food Safety and Chemical Analysis, Prof. Wacław Dąbrowski Institute of Agricultural and Food Biotechnology–State Research Institute, Rakowiecka 36 Street, 02-532 Warsaw, Poland; marek.roszko@ibprs.pl

**Keywords:** gut microbiota, dysbiosis, obesity, detoxification, BPA analogs, probiotic

## Abstract

Bisphenols, such as bisphenol A and its analogs, which include bisphenol S, bisphenol F, bisphenol AF, and tetramethyl bisphenol F, are chemical contaminants commonly found in food that raise serious health concerns. These xenobiotics can potentially have harmful effects on human health. The gut microbiota plays a crucial role in metabolizing and neutralizing these substances, which is essential for their detoxification and elimination. Probiotic supplementation has been studied for its ability to modulate the gut microbiota’s composition and function, enhancing detoxification processes. Next-Generation Probiotics (NGPs) may exhibit better properties than traditional strains and are designed for targeted action on specific conditions, such as obesity. By modulating inflammatory responses and reducing the secretion of pro-inflammatory cytokines, they can significantly improve host health. Research on NGPs’ ability to neutralize obesogenic bisphenols remains limited, but their potential makes this a promising area for future exploration. This review aims to understand the mechanisms of the chemical transformation of bisphenol through its interactions with the gut microbiota and the role of probiotics, particularly NGPs, in these processes. Understanding the interplay between bisphenols, gut microbiota, and NGPs may pave the way for strategies to counteract the negative health effects associated with daily and chronic exposure to bisphenols, which is crucial for food safety and consumer health protection.

## 1. Introduction

In recent years, chemical food contamination has become an increasingly serious problem for public health and food safety, particularly in highly industrialized countries. Exposure to various synthetic chemicals is an integral part of our lives, as they are present in many products used daily. The primary way humans are exposed to industrial chemicals is through their diet, especially by consuming contaminated foods like meat, fish, fruits, vegetables, milk, and dairy products [1]. The sources of food contamination are agriculture (pesticides), materials in contact with food (bisphenols, phthalates), anthropogenic soil and water pollution (PCB, PBDE, heavy metals, organic tins, alkylphenols), and food processing (benzo[a]pyrene) [2]. Some compounds from the group of Endocrine-Disrupting Chemicals (EDCs), such as bisphenol A (BPA) and its analogs like bisphenol S (BPS), bisphenol F (BPF), bisphenol AF (BPAF), and bisphenol B (BPB), are commonly used in the production of various food packaging materials, including plastic containers for food and drinks, as well as lining materials for cans of food and beverages [3,4]. Small amounts of EDCs migrate from packaging into food, causing daily exposure, the concentration of which depends on the country of origin, type of food, packaging, and socioeconomic status, lifestyle, and dietary habits of the consumer [1,5,6].

EDCs exert negative health effects by disrupting hormonal functions, as they can mimic or block the natural actions of endocrine hormones in the body through various known and different mechanisms. Grün and Blumberg hypothesized that some EDCs may promote fat accumulation in target cells, such as adipocytes and hepatocytes, or interfere with key metabolic processes, potentially leading to obesity and metabolic syndrome [7]. EDCs can affect the differentiation of mesenchymal stem cells (MSCs) into preadipocytes and their further maturation into adipocytes. This process is regulated by transcription factors such as CCAAT/enhancer-binding proteins (C/EBPs), which activate the PPAR-γ receptor, a key regulator of adipocyte differentiation [8]. BPA was one of the first food-related chemicals recognized as an obesogenic EDC, alongside pesticides and heavy metals. As an EDC, it can increase adipogenesis and lipid storage through interactions with nuclear receptors (NRs), particularly by activating RXR/PPARγ-dependent signaling pathways [1,9,10,11,12,13]. Notably, BPA exposure causes metabolic dysfunction in mature fat cells and triggers the production of pro-inflammatory cytokines, which, as an endocrine disruptor, can predispose individuals to obesity [8]. Research suggests that prenatal or perinatal exposure to EDCs may increase fat storage from early life stages. The obesogenic effects of certain EDCs are linked to disrupted appetite and satiety signaling, changes in food preferences [14], reduced energy expenditure, and thermogenesis in brown adipose tissue [15], as well as the promotion of inflammatory states [16]. These “environmental obesogens” represent a new, critical area of research into the impact of industrial chemicals on human obesity and metabolic disorders [17].

EDCs accumulating in the gut can significantly affect the microbiota, leading to dysbiosis, weakened immune function, and altered glucose and lipid metabolism. This may result from the fact that exposure to EDCs such as BPA can influence changes in gut morphology, villi length, proliferation, and the self-renewal of intestinal stem cells. Consequently, disturbances in the differentiation of intestinal stem cells may have negative consequences for gut homeostasis and function. Interestingly, it has been shown that these compounds can disrupt key signaling pathways and intercellular communication, which are essential for maintaining intestinal homeostasis [18]. Moreover, exposure to these compounds reduces the diversity of the gut microbiota (GM), lowers short-chain fatty acid (SCFA) levels, and contributes to gut barrier dysfunction and elevated lipopolysaccharide (LPS) levels, which consequently trigger chronic inflammation [8]. Studies confirm that prolonged exposure to obesogenic EDCs disrupts the biodiversity and metabolic activity of the GM, leading to dysbiosis and potentially chronic metabolic disorders [7,19]. EDCs are classified as microbiota-disrupting chemicals (MDCs), and the GM, through bidirectional interactions, can transform and metabolize these compounds into biologically active or inactive forms. Simultaneously, these substances can alter the abundance of different bacterial species, impacting the entire microbiome structure and disrupting the body’s homeostasis.

There is increasing evidence that probiotics, such as lactic acid bacteria (LAB), can bind to and/or degrade food contaminants, providing a safe method of removal without compromising the nutritional value of food [20]. Studies show that administering probiotics containing a mix of lactic acid bacteria and bifidobacteria after BPA exposure helped restore the normal composition of the gut microbiota, reducing the number of pathogenic species. This confirms their effectiveness in alleviating the toxicity caused by EDCs. Additionally, supplementation with these strains increased the number of goblet cells, which produce acidic mucins, thereby protecting the intestinal epithelium [21]. However, to effectively counter disrupted microbial profiles and specific imbalances caused by exposure to chemical food contaminants, dedicated and integrated preventive and therapeutic measures are essential. In this context, the search for NGPs becomes crucial [22]. Mitigating the effects of chemical contamination exposures is vital, as chronic exposure to these substances can lead to the development of metabolic diseases and chronic inflammatory states. Currently, *Akkermansia muciniphila* and *Faecalibacterium prausnitzii* are among the candidates for NGPs. According to recent reports, they have promising potential in combating diseases resulting from inflammation-based dysbiosis caused by exposure to chemical food contaminants. This is due to their ability to interact with the host’s immune system, helping to maintain a healthy immune response and contributing to gut homeostasis, which is crucial in preserving the integrity of the gut barrier and preventing toxins or chemicals from entering the bloodstream [23]. Although research on their ability to detoxify dietary xenobiotics is still limited, their anti-inflammatory and dysbiosis-ameliorating properties suggest that they may play an important role in this process [24,25].

In this review, we have summarized current knowledge on the mechanisms underlying the biodetoxification of the obesogenic effects of dietary chemicals by the GM gut microbiota and the potential role of probiotic supplementation in this process. In particular, we discussed the potential implications of these mechanisms in mitigating human exposure to BPA and its analogs (BPS, BPF, BPB, BPAF, TMBPF), which are commonly used in the food industry. We provided insights into future research directions in this field. Understanding the interactions between food chemicals and probiotics could open new avenues for developing strategies to alleviate the harmful health effects associated with chronic, everyday dietary exposure.

## 2. The Bisphenols Used in the Food Industry

### 2.1. Bisphenol A

Bisphenol A (BPA, 4,4′-methanediyldiphenol) is the most well-known EDC utilized in the food industry. BPA is highly soluble in water (120 mg/L at 25 °C) and has a moderate bioaccumulation potential according to its chemical–physical properties. The commercial production of BPA in the United States of America (US) began in 1957, and it began one year later in Europe [15]. BPA is a chemical produced in large quantities for use primarily in the production of polycarbonate plastics. It is commonly found in products intended for everyday use such as plastic water bottles, reusable food containers, thermal paper, dental materials, and medical equipment [26,27,28]. Due to its temperature resistance and transparency, it is used to produce optical media, electronic equipment, food containers, and reusable water bottles. Moreover, it is produced as a monomer ingredient in polycarbonate plastics, which are used in drink containers and food packaging. High temperatures and exposure to acidic or alkaline solutions can enhance the process of BPA leaching from coatings and synthetic materials. Cao et al. [29] showed that the likely source of BPA in beer products is the coating of the cans, from which BPA migrates into the contents, mainly due to hydrolysis caused by heat, acids, or alkaline conditions. BPA is also used to produce epoxy resins, which are used as coatings for metal cans that have direct contact with food and drinks [29].

Given its widespread use in food packaging materials, concerns about BPA migration into food and beverages have been raised. Although BPA can migrate from plastic into food and beverages in very small amounts, chronic intake and exposure have been responsible for its detectable levels in human serum and urine. BPA negatively impacts metabolism by disrupting the synthesis, secretion, transport, binding, activity, and metabolism of estrogenic hormones [30]. In developing countries, concentrations of BPA in human serum and plasma range from 0.3 to 4.4 ng/mL (1.3 to 19.4 nM) [31]. In 2005, the Centers for Disease Control and Prevention (CDC) reported that in a population of 394 adult Americans, BPA was found in 95% of urine samples; the average BPA level in urine for men and women was 1.63 ng/mL and 1.12 ng/mL, respectively [32]. Moon et al. [33] demonstrated a significant correlation between urinary BPA levels and an increased risk of obesity in adult Koreans. BPA levels were significantly higher among obese adults than among non-obese adults [33]. In addition, a link between elevated BPA levels in urine and the occurrence of cardiovascular diseases has also been documented [34].

In adults, the biologically active form of BPA, known as unconjugated BPA, is quickly conjugated in the liver by UDP-glucuronosyltransferase (UGT) and subsequently eliminated through bile or urine, with a half-life of around 5.3 h [35,36,37]. This rapid excretion has been the foundation for some public health authorities worldwide to provide reassuring safety assessments and declarations. However, in various tissues, especially the lungs, liver, kidneys, and placenta, enzymes such as β-glucuronidase and steroid sulfatase, which are present at detectable levels, can de-conjugate BPA, thereby reactivating its biologically active form [29,38]. During pregnancy, the conjugated form of BPA passes through the placenta, where it undergoes deconjugation, leading to fetal exposure in utero. The fetus is particularly vulnerable due to the absence or reduced activity of UGT, especially during the first two trimesters [39].

This can also lead to the bioaccumulation of a portion of BPA following exposure. Studies have reported that BPA exposure during pregnancy and early childhood is as-sociated with numerous fetal and perinatal adverse effects, including reduced growth velocity and preterm birth [39]. BPA exposure can induce epigenetic changes such as DNA methylation, post-translational modifications of histones, and effects on non-coding RNAs, which can persist throughout the life of an individual. These lasting changes can result in negative health outcomes, including neural and immune disorders, infertility, and late-onset complex diseases like cancer and diabetes. Consequently, the migration of BPA into baby food or infant formula has been prohibited (Directorate-General for Health and Food Safety, European Commission, 2020). Since 2018, the European Union (EU) has mandated that products containing BPA be classified and labeled as toxic for reproduction [40]. On 9 February 2024, the European Commission released a draft regulation aimed at prohibiting the use of BPA in food contact materials, including plastics and coatings used on metal cans. The basis for this initiative was the BPA risk assessment conducted by the European Food Safety Authority (EFSA) in 2023 (European Commission, 2024).

Numerous studies have confirmed the link between long-term exposure to BPA and increased obesity in humans [41,42,43]. BPA affects energy metabolism and food intake by directly increasing the number of fat cells and promoting fat storage in existing adipocytes. It alters the body’s energy balance in favor of calorie storage by influencing the basal metabolic rate and the hormonal regulation of appetite and satiety. In vitro studies on the effect of BPA on the differentiation of mouse and human stem cells into mature adipocytes showed an increase in the expression of genes related to adipogenesis. Additionally, disruptions in metabolic functions, an elevated expression of pro-inflammatory cytokines, and an increased accumulation of visceral fat were observed [43].

Moreover, BPA impacts fat tissue metabolism by modifying the function of glucocorticoid receptors (GRs). By binding to GRs, BPA increases lipid accumulation and adipogenesis through the upregulation of genes involved in fat storage, such as lipoprotein lipase. These mechanisms contribute to the development of obesity and its associated health complications. Sargis et al. [44] confirmed this in their research, showing that BPA stimulates adipogenesis in 3T3-L1 mouse cells via GR activation. BPA has also been linked to various human diseases, including diabetes, reproductive disorders, cardiovascular diseases, birth defects, and breast cancer [45].

Due to these harmful effects on human health, several countries, including Canada (2009), the US (2010), the EU (2011), and China (2011), have banned the use of BPA in products intended for infants [46]. A prospective birth cohort study in Korea found that BPA exposure was negatively associated with the growth of fetuses and children from the fetal stage up to 72 months after birth. For exclusively breastfed infants, breast milk was the main source of BPA exposure. However, the impact of exposure to BPA alternatives (such as BPS, BPF, and BPAF) through breastfeeding on infant growth remains poorly understood [46].

Reina-Peréz et al. [28] investigated the *in vitro* effects of BPF and BPS on the adipogenesis of human adipose-derived stem cells (hASCs), which were exposed to different doses of the compounds (0.01, 0.1, 1, 10, and 25 M). hASCs exposed to BPF or BPS showed a linear dose response in terms of intracellular lipid accumulation and adipogenic gene expression. This indicates that both BPF and BPS promote human adipogenesis by interfering with adipocyte developmental programming and increasing intracellular lipid accumulation, which affects their obesity potential [28]. Hugo et al. [11] demonstrated in their research that BPA at doses of 0.1 and 1nM suppresses the release of adiponectin, a hormone specific to adipocytes that increases insulin sensitivity, reduces tissue inflammation, and protects against metabolic syndrome. BPA also has a proangiogenic effect on the human endothelium, suggesting that this is a viable target for BPA [47].

### 2.2. BPA Analogs

As a result of public concern demanding BPA-free products, BPA is being replaced by a variety of BPA analogs (BPs), such as bisphenol S (BPS), bisphenol F (BPF), bisphenol B (BPB), bisphenol AF (BPAF), bisphenol E (BPE), bisphenol Z (BPZ), and tetramethyl bisphenol F (TMBPF). BPS and BPF are currently widely used as substitutes in a variety of consumer and industrial products [40,48]. Thus, humans are increasingly exposed to these structural BPA analogs, but available data on their presence in the environment are more limited than for BPA. Nonetheless, the occurrence of BPs was reported in river- and seawater, wastewater, and in sediment [49]. Additionally, several studies have indicated that BPF and BPS are more resistant to environmental degradation compared to BPA [50,51].

Regarding human biomonitoring for exposure to chemicals, BPS and BPF were detected in 78% (0.13 ng/mL) and 55% (0.08 ng/mL) of urine samples collected in 2009–2012 from adults in the US [52]. In turn, according to results from the US National Health and Nutrition Examination Surveys (NHANESs), in 2013–2016, the levels of BPF and BPS estimated in the urine samples of children (n = 1831) from 8 to 19 years old were 55.2% and 87.8%, respectively [53]. In addition, these BPs were widely detected in the serum of pregnant women and in the breast milk of Chinese women [47].

Although the chemical structures of BPs are similar to that of BPA, the safety of these products was not tested before they were marketed [54]. Recent *in vitro* and *in vivo* studies have revealed that many of these BPs are not as safe as previously thought, exhibiting similar or even stronger toxic and estrogenic effects compared to BPA [55,56,57]. This is particularly concerning due to their impact on obesity. Research has confirmed that BPA analogs can act as obesogens—compounds that promote weight gain.

**Figure 1:**
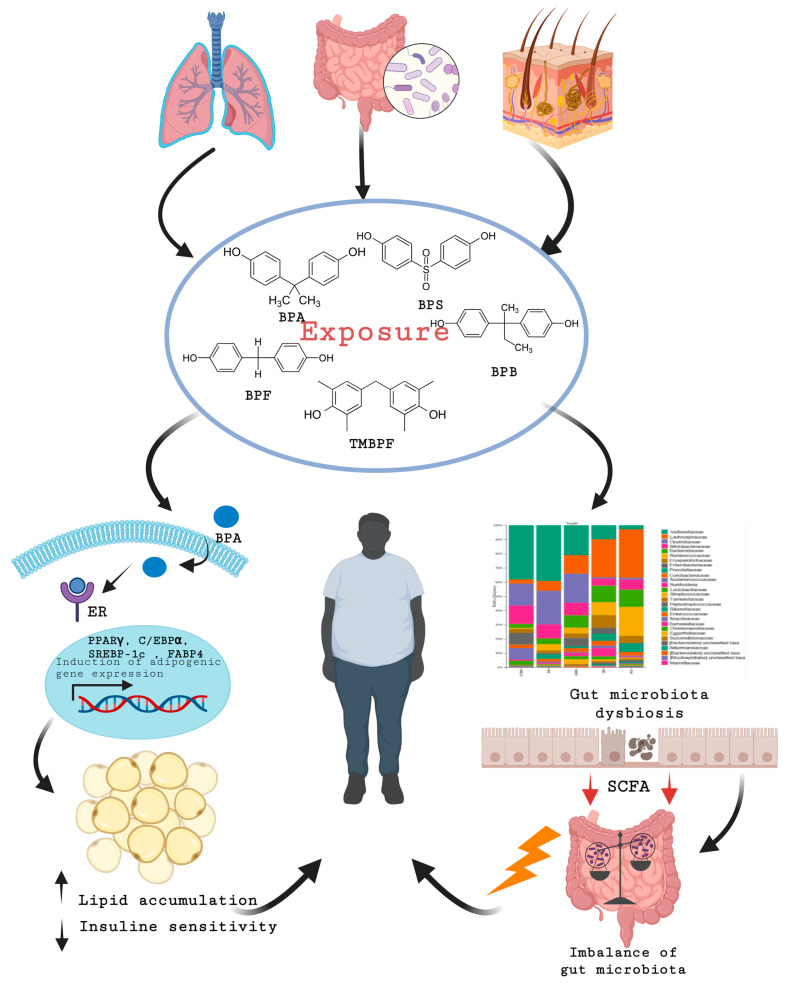
Exposure pathways of BPA and its analogs and the disorders that results. The graphic illustrates that exposure to BPs can occur through the digestive system, respiratory system, and skin. It also shows the impact of BPs on the process of adipogenesis in ASCs (human adipose stromal cells) and disruptions in the taxonomic composition of the gut microbiota. A possible mechanism of bisphenol action involves BPs’ penetration into ASCs, binding to the estrogen receptor (ER), translocating to the cell nucleus, and increasing the expression of adipogenic genes such as PPARy (Peroxisome Proliferator-Activated Receptor), C/EBPα (Enhancer-Binding Proteins), SREBP-1c (Sterol Regulatory Element-Binding Protein-1c), and FABP4 (Fatty Acid Binding Protein 4). This mechanism is associated with adipogenesis, which accelerates the maturation of ASCs into mature adipocytes, promotes obesity, and reduces insulin sensitivity. Another disorder resulting from bisphenol exposure is the dysbiosis of the gut microbiota, characterized by a decreased production of SCFA, leading to imbalances in the gut microbiota, chronic inflammation, and obesity.

Exposure to BPs can occur through the digestive, respiratory, and dermal routes. Once inside fat cells, BPs activate estrogen receptors and increase the expression of genes related to adipogenesis, stimulating the differentiation of preadipocytes into mature adipocytes. This leads to excessive fat accumulation and reduced insulin sensitivity. Additionally, bisphenols disrupt metabolism and hormonal regulation, affecting key hormones involved in weight regulation, such as insulin, leptin, and estrogen. They may also cause gut dysbiosis, leading to chronic inflammation and the further development of obesity [43,52,58,59,60] (Figure 1). Despite their similar chemical structures and presumed BPA-like biological effects, many bisphenols remain largely unregulated. In Europe, BPS is permitted in plastic food containers with a migration rate limit of 0.05 mg/kg, while other BPs are not subject to regulation.

In this chapter, some BPA substitutes widely used in the food industry are highlighted and their possible impact on human health is summarized.

#### 2.2.1. Bisphenol S (BPS)

Bisphenol S (BPS, bis (4-hydroxy phenyl) sulfone) was one of the first analogs of BPA widely used in canned foodstuffs, food cartons, luggage tags, newspapers, epoxy and phenolic resins, and as a bleach stabilizer in cleaning agents [58]. BPS is used as a starting monomer in the synthesis of polyether sulfone, which is employed in the production of baby bottles and children’s tableware [61]. Moreover, BPS is also commonly used in thermal receipt papers marketed as “BPA-free” [62]. High concentrations of BPS were found in 62% of thermal paper receipt samples from Italy [63] and all paper samples from the US, Japan, Korea, and Vietnam [64]. Moreover, a relatively high concentration of BPS was found in thermal food labels, like price tags and stickers from Canada [52]. According to ECHA, 1000 to 10,000 million metric tons of BPS are produced or imported yearly into the European Economic Area [53]. Recently, it has been reported that BPS has become the main BPA replacement [33,53]. In Europe, BPS was detected in wastewater samples and canned food. Regarding food/foodstuffs, BPS was detected in dairy products, meat and meat products, vegetables, canned foods, and cereals [63,64]. BPS, together with other BPs, was found in the majority (78%) of canned foods from China. A high total concentration of BPs, including BPS, was found in beverages, fish and seafood, fruits, and condiments [63].

BPS exposure frequently occurs through ingestion, inhalation, and dermal contact [65]. In human biomonitoring trials, BPS was detected in 89.4% and 81.0% of urine samples from US and Asian populations, respectively, including adults and children [47,53,64]. Wang et al. [66] showed that the concentration of urinary BPS, similar to BPA, was higher in males than females. In addition, a significantly greater level was observed in the urine of young adults (15–30 years old) than in children (0–15 years old). In turn, Tang et al. [67] reported that in urine samples collected between 2012 and 2017 from the Australian population, BPA and BPS were predominant, with concentrations of 2.50 µg/L and 0.64 µg/L, respectively. BPS was detected in adults’ plasma from China with a detection rate of 56% and with a mean concentration of 0.15 ng/mL [47], and a greater level was observed in the young than in older adults [64]. In two studies of cashiers handling thermal receipts, significantly higher serum and urinary BPS concentrations were detected than those in non-cashiers [68,69].

A longitudinal birth cohort study of 190 Chinese women demonstrated that breast milk can be the first line of BPS exposure in infant; in breast milk samples from their mothers, the BPS concentration was 0.19 ng/mL [47]. In turn, a study conducted by LaPlante et al. [70] showed that BPS exposure in pregnant and lactating mice was associated with limited milk production, which may suggest alterations in mammary gland function [68]. Liu et al. [71] showed that BPS can cross the human placenta because it was detected in maternal and cord blood samples of Chinese women, with a range of 0.03 to 0.07 µg/L. A similar result was also reported by Connors et al. [72], who showed that BPS can cross into the placenta and accumulate in the fetus to a greater extent than BPA. On the other hand, a study conducted on pregnant sheep demonstrated that BPS crosses the placenta with lower efficiency than BPA, but its accumulation in the fetus is greater due to its slower clearance. Therefore, exposure to BPS during pregnancy can lead to placental dysfunction and can also result in reproductive and metabolic disorders in the progeny [73]. Recent reports indicate that exposure to BPS, even at very low levels, can impact brain function. In female rats, exposure to concentrations comparable to human exposure levels of BPS (2 µg/kg) led to altered maternal behavior during pregnancy [74].

The systematic analysis of the available scientific literature data including *in vitro*, *in vivo*, and human epidemiological trials of BPS shown that it has a lower cytotoxic and genotoxic activity compared to BPA and other analogs [55,75]. However, it has been reported that the estrogenic effect of BPS is higher than that of BPA [62,76]. For example, BPS exhibits higher hormonal activity due to its polarity and the presence of a sulfonyl group in its structure [77,78]. A comprehensive review of the literature showed that BPS bound to estrogen receptors (ERs) and affected estrogenic and antiandrogenic activity in both *in vitro* and *in vivo* models in a manner similar to BPA [62,79]. For example, studies in zebrafish, rodents, and human cell culture models show that BPS has endocrine-disrupting activity [80,81]. Furthermore, studies in zebrafish, rodents, and human cell culture models show that BPS and BPF have similar endocrine-disrupting activities. In zebrafish, despite species-specific differences in ER affinity and specificity, BPF and BPS have estrogenic activities similar to BPA [25,82,83]. BPS also exhibits a potency comparable to that of estradiol in membrane-mediated pathways crucial for cellular processes such as proliferation, differentiation, and apoptosis [62].

Research on the impact of BPS on the adipogenesis process in primary human preadipocytes has shown that the fold change in lipid accumulation growth and adipogenic expression levels induced by BPS appears to be higher than that observed previously for BPA [62,79]. BPS was first described as a compound that causes obesity at low doses and after post-birth and chronic exposure in male mice, where it contributed to the exacerbation of obesity caused by a high-fat diet. Halogenated BPA analogs, as well as BPS, have been shown to be more potent activators of PPARγ [79] and stronger promoters of adipogenesis in 3T3-L1 preadipocytes compared to BPA. Perinatal exposure to BPS also led to obesity in mice [78]. A longitudinal birth cohort study found a significant association between BPS exposure and obesity in children aged 6–19, while BPA and total bisphenol levels did not show significant associations. Epidemiological studies also suggest a link between BPS’s presence in urine and the development of obesity and diabetes [28]. In vivo and *in vitro* studies showed that exposure to BPS may lead to obesity via an increase in preadipocyte differentiation and promotion of fat accumulation in adipocytes [58,84]. Moreover, like BPA, BPS has been linked to the development of breast cancer, as it induces the proliferation and migration of clonal MCF-7 cells [58].

#### 2.2.2. Bisphenol F (BPF)

Bisphenol F (BPF, 4,4′-dihydroxy diphenyl methane) is used to produce epoxy resins and coatings, particularly in systems requiring increased thickness and durability such as pipelines and structural adhesives. BPF epoxy resins are also used in many consumer products such as food packaging. BPF is also used as a developer in thermal paper, including products marketed as “BPA-free” paper [84]. Interestingly, the highest concentration of BPF found was 1130 ng/g, which was found in a sample of mustard and ginger [76].

BPF is widely prevalent, as confirmed by studies analyzing urine samples collected as part of the NHANES in the years 2013–2014. The study included urine samples from both adults and children in the US. However, in their research, the average concentration of BPF in children’s urine was slightly higher than that of BPS [53].

In comparison to BPS, the association between BPF and obesity is less clear. On the one hand, similar to BPS, a correlation has been established between the presence of BPF in urine and the occurrence of obesity and diabetes. The detection of BPF was also linked to abdominal obesity [84]. BPF showed a positive association with overweight and an overall increase in BMI *z*-score [85]. In the studies conducted by Liu et al. [86], it was observed that BPA and BPF show stronger associations with obesity in boys than in girls. Similar findings were reported by Gajjar et al. [87], indicating an increase in waist circumference in girls after 8 years of exposure to BPS, with no noticeable changes in boys. However, it is important to emphasize that for a comprehensive understanding of the impact of gender differences on the alteration of people’s susceptibility to the adverse effects of exposure to BPs, further research is recommended.

On the other hand, Drobna et al. [88] conducted a study comparing the impact of BPA, BPS, and BPF on the differentiation of murine 3T3-L1 preadipocytes. They also carried out an *in vivo* study on male mice exposed to three different doses of BPF for 12 weeks. It was observed that BPF had no effect on lipid accumulation and, moreover, that it reduced the expression of several adipogenic markers. Additionally, BPF resulted in a smaller increase in body weight in male mice compared to the control group [88,89].

The effects of obesity and the metabolic disorders associated with BPF may result from diverse pathophysiological mechanisms. Rochester and Bolden [62] conducted a meta-analysis including 32 studies (25 *in vitro* and 7 *in vivo*), showing that BPF has similar cytotoxicity and estrogenic, antiestrogenic, androgenic, and antiandrogenic activity to BPA, as well as a similar potency. Studies suggest that BPF induces obesity effects primarily through the activation of estrogenic or androgenic actions. However, the reported mechanisms are not limited solely to these aspects; BPF may also lead to significant changes in the gene expression of various markers associated with adipogenesis [84].

In addition to their estrogenic and androgenic effects, the obesogenic action of BPA and its analogs, including BPF, has been attributed to disruptions in the upregulation of key genes associated with adipogenesis, such as PPARγ and C/EBPα [28]. This was corroborated by studies where, in 3T3-L1 preadipocytes incubated with BPA, BPS, or BPF at a concentration of 20 µM for 10 days, an increase in the levels of the adipogenic markers PPARγ and C/EBPα was observed under the influence of all three BPs [90].

#### 2.2.3. Bisphenol B (BPB)

Bisphenol B (BPB, 2,2-bis(4-hydroxyphenyl)butane) is an analog that is very structurally close to BPA. It differs from BPA only by an additional methyl group on its central carbon atom [91]. BPB is used to produce phenolic resins in some countries as a substitute for BPA. In the US, BPB is registered as a direct additive for food contact coatings and polymers [91], but it is not registered under the European Chemical Agency (ECHA) regulation for manufacture and use as a chemical substance in Europe (EU Regulation CLP 1272/2008/WE). The ECHA has identified BPB as substance of very high concern (SVHC) for its activity as an EDC towards human health and the environment (European Food Safety Authority, EFSA, 2021). BPB has been detected in different environmental media [91], indoor dust [64], and foodstuffs [92]. BPB has been detected in food samples in several studies mainly conducted in Europe and Asia. It is found in canned and non-canned food products [93], but also in egg and oil from Spain [94], as well as in commercial milk samples. For instance, in 95 food samples from Italian supermarkets, the concentration of BPB was 21.4% [92], but recent results showed that is no risk of exposure to BPB for the Italian population [95]. In twenty-three samples of canned legumes from popular market brands in Italy, BPB was not detected compared to BPA, which was found in 91% of tested samples in a concentration range of 1.51–21.22 ng/mL [95]. On the other hand, there are very limited data on human exposure to BPB. An Italian study reported the presence of BPB in serum from healthy and endometriotic women at a mean concentration of 5.15 ng/mL, greater than BPA concentrations (mean: 2.91 ng/mL). Positive results for BPB in urine samples have also been reported by Cunha and Fernandes [92].

A growing number of studies show that BPB possess estrogenic and/or antiandrogenic activities that are similar to or greater than BPA. Moreover, compared to BPA, BPB has much higher acute toxicity [91,96] and is more resistant to biodegradation in aquatic environments [51]. In a meta-analysis by Serra et al. [91], BPB has exhibited a strong potential to bind to ERα and ERβ, including in human, rat, and mouse *in vitro* models [91]. In addition, the estrogenic response induced by BPB showed a higher affinity than BPA towards ERα [45,86] and towards ERβ [45]. In turn, recent *in vivo* studies confirmed the adverse effects of BPB on the male reproductive system in rodents and fish [57,97]. In male Sprague Dawley rats, BPB showed a toxic effect on the testes and spermatogenesis via the induction of oxidative stress, leading to reproductive dysfunction [57,97]. In zebrafish, BPB was demonstrated to significantly reduce the fecundity of adult fish exposed to it for 21 days and to decrease embryo hatching and the survival of the F1 generation. In turn, the antiandrogenic action of BPB was demonstrated in an *in vitro* model of steroidogenesis and *in vivo* in fish and rodents [97,98]. In addition, Serra et al. [91] reported that the estrogenic and antiandrogenic activity of BPB were similar to or even greater than the hormonal activity of BPA. Moreover, an increase in BPB’s estrogenic ability after incubation with liver S9 fractions has also been reported by Hashimoto et al. [99]. Using a 3T3-L1 mouse model of adipocytes, it has been reported that BPS, similar to BPA, may increase insulin resistance by inhibiting adiponectin production in cells, resulting in a reduction in adiponectin secretion [59,100]. Kidani et al. [100] determined the impact of BPA and BPB at a concentration of 80 µM on adiponectin secretion and showed that BPB significantly reduced intracellular adiponectin levels compared to BPA (−89% and −57%, respectively). In addition, the strong effect of BPB (5 µM) on *in vitro* lipid accumulation in 3T3-L1 cells has been reported by Ramskov Tetzlaff et al. [59]. Thus, on 9 March 2021, the French Agency for Food, Environmental, and Occupational Health & Safety (ANSES) issued a press release announcing its proposal to classify BPB as a substance of very high concern (SVHC) under the EU’s REACH regulation, similar to BPA, due to its endocrine-disrupting properties (ANSES, 2021).

#### 2.2.4. Bisphenol AF (BPAF)

Bisphenol AF (BPAF, 4-[1,1,1,3,3,3-hexafluoro-2-(4-hydroxyphenyl)propan-2-yl]phenol) is a structural analog of BPA where both methyl groups are substituted by trifluoromethyl groups [101]. BPAF is primarily employed as a cross-linking agent during the fabrication of fluoroelastomers and serves as a monomer in the manufacturing processes of polycarbonates, polyamides, and polyesters. The US National Toxicology Program has approved BPAF for thorough toxicological evaluation. In a study conducted by Yang et al. [102], approximately 30% of urine samples from 94 volunteers residing near a production facility in China exhibited a presence of conjugated BPAF, with a geometric mean urine concentration of 0.018 µg/L. Meanwhile, BPAF was identified in less than 3% of 616 urine samples collected from US adults, with concentrations ranging from <0.10 µg/L to 0.12 µg/L [103].

Several *in vitro* studies showed that BPAF exhibits activity as an agonist of ERs via binding to both ERα and ERβ and acting as an EDC [103]. Cohen et al. [43] investigated the impact of BPA and its analogs, including BPAF, on adipogenesis in human adipose tissue-derived stem cells *in vitro*. They found that a 0.1 nM concentration of both BPA and BPAF significantly impacted adipogenesis, with effects similar to E2 (estradiol), and, at higher doses, they were associated with reduced adipogenesis and increased apoptosis. This suggests potential toxicity even at environmentally relevant low doses.

Furthermore, BPAF activates inflammatory signaling pathways that degrade metabolic activity in human adipocytes [43]. Mu et al. demonstrated that, in zebrafish embryos, BPAF induced ER more significantly than BPA, reaching a concentration equivalent to 1% LC50. Li et al. [104] found that BPAF-G (bisphenol AF glucuronide), a metabolite of BPAF, did not exhibit estrogenic activity. This implies that the generation of BPAF-G might serve as a potential defense mechanism by the host against the toxic form of BPAF. Escrivá et al. [101] indicated that BPAF exposure can cause impaired fertility in women and men by disrupting estrogen and androgen signaling.

#### 2.2.5. Tetramethyl Bisphenol F (TMBPF)

Tetramethyl bisphenol F is a synthetic chemical compound with the trade name valPure V70 that was recently developed as an analog of BPA. The Valspar company (Minneapolis, Minnesota, United States) received a Bronze Metpack 017 Innovation Award for the production of a new generation of valPure V70 BPA-free coatings for lightweight metal packaging. TMBPF has been sold in the US since 2017 and has been used in more than 22 billion cans. TMBPF has technical properties, durability, and polymer integrity similar to its parent compound and was developed as a coating for food contact in metal cans for drinks and food (Valspar Packaging Award-Winning Innovation, 2017). Moreover, it has been reported that TMBPF has limited migration from polymers, below the detectable limit of 0.2 µg/kg (0.2 ppb) [105,106,107].

There are conflicting results regarding the estrogenic and antiandrogenic activity of TMBPF compared to other bisphenols, both *in vitro* and *in vivo* [PP]. According to a study conducted by Soto et al. [106], TMBPF did not exhibit estrogenic activity or act as an estrogen antagonist in the estrogen receptor transactivation assay, nor did it affect the development of mammary glands in rats. In contrast, a study conducted by Szafran et al. [107] showed that TMBPF has both antiestrogenic and antiandrogenic effects in human cell lines such as HeLa, breast cancer (MCF7), and prostate cancer (LNCaP) cell lines using several highly efficient systems based on human cells. Maffini and Canatsey [108] showed that TMBPF has a different toxicological profile compared to other bisphenols. TMBPF showed a cytotoxic effect at both low and high doses of 0.01 and 10 uM. Harnett et al. [55] have demonstrated that TMBPF exhibits a 100-fold greater cytotoxicity than BPA in human stem cells and activates apoptosis through non-estrogenic pathways. Cohen et al. [43] have shown that TMBPF exhibits reduced adipogenesis in human stem cells, which can be explained by the high cell death rate. These reports emphasize the greater potency of TMBPF compared to BPA [10,43]. Currently, there are insufficient data to assess the effects of BPAF on human health. Further research is necessary to better understand the potential risks that TMBPF may pose to human health.

## 3. The Impact of Bisphenols on Gut Microbiota and Their Obesity Risk

According to the latest report, by 2035, over half of the world’s population, which is more than 4 billion people, will be affected by overweight or obesity if significant action is not taken. The 2023 World Obesity Federation Atlas predicts that within the next 12 years, 51% of the global population will be obese or overweight. The report indicates that obesity rates are growing particularly fast among children and in lower-income countries. Due to easy access to high-calorie processed food, the prevalence of overweight and obesity is steadily increasing. Experiments on germ-free animals have provided evidence suggesting the involvement of bacteria in obesity. This has encouraged scientists to conduct further research exploring the role of the GM in the development of obesity [10,109,110].

The GM is a community of microorganisms living in the digestive tract. The intestines are colonized by approximately 10^14^ microorganisms and over 1000 species of bacteria [20,111]. However, there is a distinct spatial difference in the composition and number of microorganisms in the digestive tract [82]. Metagenomic analyses have shown that a healthy intestine is dominated by *Firmicutes* and *Bacteroidetes*, with smaller amounts of *Actinobacteria* and *Proteobacteria* [14].

The richness of the GM is modulated by diet, environment, stress, and genetic factors. Eating habits and a high-fat, low-fiber diet typical of Western countries have negatively impacted the GM’s composition and function. Cani et al. [10] showed that a high-fat diet increases the probability of endotoxemia [10,112]. In recent years, the alteration of GM composition has been associated with the consumption of highly processed and ready-to-eat packaged food, exposing consumers to toxic contaminants applied in the food manufacturing process. Changes in GM structure lead to dysbiosis associated with increased intestinal permeability and immune-mediated inflammation, which promotes the development of metabolic diseases such as obesity [113].

Recent research has demonstrated that BPA exposure induces dysbiosis in the GM and disrupts the metabolomic profile associated with the microbiota, leading to disturbances in host metabolite homeostasis. This dysbiosis is particularly evident in obese individuals, manifesting as a reduction in bacterial diversity and an elevated *Firmicutes* to *Bacteroidetes* ratio compared to lean individuals [109].

Researchers have conducted studies on the impact of BPA and its analogs in various animal models, including rodents [114,115], zebrafish [116], rabbits [117], and dogs [118]. Ley et al. [119] found that obese mice exhibited a 50% reduction in *Bacteroidetes* and a proportional increase in *Firmicutes* compared to lean mice. Interestingly, even when both groups were fed the same diet, the results suggest that obesity may influence GM diversity. Javurek et al. [114], using a murine model, showed that parental exposure to BPA was able to disrupt the microbiota structure in non-exposed offspring. Changes in microbiota composition were dependent on the generation and sex of the tested mice. Significant increases in *Akkermansia*, *Mollicutes*, *Prevotellaceae*, *Bacteroides*, *Erysipelotrichaceae*, *Methanobrevibacter*, and *Sutterella* levels were observed in both parents and monogamous mice, including females and males, when exposed to BPA or ethinyl estradiol, from preconception to weaning. These genera have been associated with inflammatory bowel disease, obesity, and metabolic disorders in humans [114]. Additionally, male mice exposed to BPA exhibited an increase in their relative abundance of *Prevotellaceae* compared to females. Age- and gender-related changes in the GM in response to BPA exposure were also reported by Lai et al. [115]. Using the non-obese diabetic (NOD) mouse model, the authors demonstrated significant differences in microbial communities between adult and juvenile females. BPA exposure in juvenile females led to an increase in *Turicibacter*, *Oscillospira*, *Ruminococcus*, *Jeotgalicoccus*, and *Lachnospiraceae*, increasing their risk of developing type 1 diabetes [115].

In a rabbit model, Reddivari et al. [117] revealed that perinatal exposure to BPA significantly reduced *Ruminococcaceae*, *Oscillospira* sp., *Odoribacter* sp., and their metabolites, such as SCFAs. In offspring, the elevated level of serum LPS was correlated with a significant increase in *Methanobrevibacter* sp. abundance, leading to increased gut permeability and colonic and hepatic inflammation. Dogs fed with canned food showed a three-fold increase in serum BPA content, disrupting their GM composition. A higher serum BPA concentration was positively correlated with increased counts of *Bacteroidetes ovatus*, *Prevotella* spp., *Ruminococcus* spp., and *Cetobacterium somerae* bacteria and negatively with the counts of *Bacteroides* spp., *Streptophyta, Erysipelotrichaceae*, and *Flexispira* spp. [118].

## 4. The Host’s Gut Microbial Metabolism of Bisphenols

### 4.1. Host Metabolism

Food, with its diverse dietary compounds, environmental chemicals, pollutants, food chemical contaminants, and medications, is considered a source of xenobiotics for the human body [120]. human exposure to xenobiotics through dietary consumption is associated with their bioaccumulation in both plants and animals, as well as their transfer from packaging materials that come into contact with foodstuff [121,122]. Their accumulation in human tissues and/or organs depends on many factors, such as their route, dose, exposure time, ability to bind ligands in host cells, and species sensitivity [20]. Poorly absorbed chemicals that are moved by peristalsis after ingestion are transported from the small intestine to the large intestine, where they are exposed to the GM [120]. Compounds readily absorbed via oral ingestion traverse the proximal segments of the gastrointestinal tract and enter the small intestine, where potential modifications by host enzymes may occur prior to their translocation to the liver via the hepatic vein [123]. Intravenously administered compounds bypass this metabolism and are introduced into the systemic circulation. These chemicals are eventually further metabolized and/or excreted, either through the bile duct into the intestinal lumen or via the kidneys into the urine [124]. Most xenobiotics are nonpolar and are absorbed in the digestive tract and transported to the liver through the hepatic vein for their detoxification.

Hepatic metabolism involves three phases: (1) activation reactions which occur through oxidation, reduction, or hydrolysis in phase I, where enzymes like cytochrome P-450 (CYP450), carboxylesterases, and flavin monooxygenases (FMOs) play a key role; (2) phase II, which is dominated by transferase enzymes, including uridine-5-diphospho-glucuronosyltansfereases (UGTs), and sulfotransferases (SULTs), attaching glucuronyl, methyl, sulfonyl, acetyl, and glutathionyl onto xenobiotics or phase I metabolites attaching moieties to xenobiotics, reducing their toxicity; and (3) phase III, which involves the transport of compounds without further transformations, leading to their excretion by the kidneys or in bile. In phase IV, the gastrointestinal microbiota further metabolizes these compounds [125,126].

The exact reasons why some chemicals are excreted through bile are not completely understood. Our current understanding posits that compounds characterized by a diminutive molecular weight (<325 kDa) exhibit suboptimal excretion through bile, whereas those with a greater molecular weight (>325 kDa) tend to be easily excreted through bile in humans [123]. Xenobiotic metabolism involves converting nonpolar compounds into hydrophilic ones with a larger 26 kDa size, which increases their elimination from the body [120,123]. Due to its small structure and lipophilic nature (logP of 3.4), BPA has the ability to cross cellular barriers and accumulate in various human and animal tissues. This can compromise physiological functions and lead to harmful effects on health [127].

**Figure 2:**
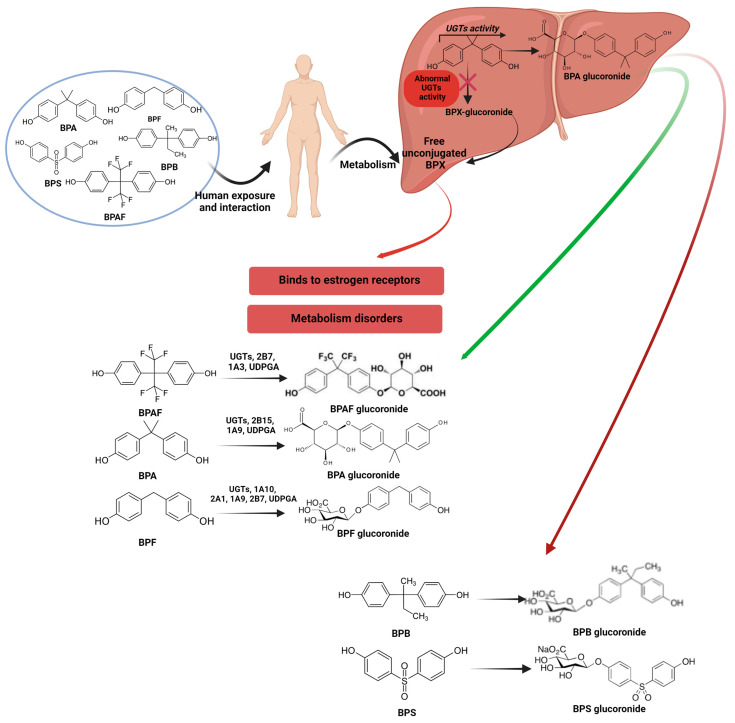
The hepatic metabolism of BPs and disruption of liver enzyme activity. The diagram illustrates the liver metabolism of BPs, which involves key liver enzymes from the Uridine 5 diphosphate glucuronosyltransferase (UGT) and sulfotransferase (SULT) families. This process aims to reduce the toxicity of these compounds and eliminate their hormonal activity, with the exception of BPB glucuronide and BPS glucuronide, which still exhibit estrogenic activity after liver metabolism. In pathological conditions such as obesity and diabetes, abnormalities in UGT activity lead to an increase in unconjugated BPs in the body, which resemble beta-estradiol and contribute to metabolic pathway disturbances and interactions with estrogen receptors.

In humans, after digestion, BPs are largely detoxified by phase II conjugating enzymes, including UGTs and SULTs [128], mainly driven by the glucuronidation of active BPs into biologically inactive BPs-glucuronide (BPs-G) (Figure 2). BPs-G and BPs-sulfate (BPs-S) are rapidly excreted via urine and feces, with a resulting half-life of <12 h [129]. For example, in humans, BPA is eliminated as BPA-G within 6 h, but considering its widespread application and chronic exposure in the general population, BPA concentrations remain persistent [122]. BPs-G is the main compound excreted in urine [119,130], but several studies confirmed the presence of sulfate conjugates of BPA and its analogues in urinary samples [128,131]. Gerona et al. [131] showed that 15% of BPA forms recovered in the urine of pregnant women were BPA-sulfate. In an *in vitro* study, Le Fol et al. [132] reported that in the human HepaRG cell line, 85.8% of BPS was conjugated to BPS-S and 10.5% to BPS-S. Recently, Khmiri et al. [128] confirmed that BPS is rapidly converted to its BPS-G form and eliminated via the urine tract after oral exposure. In addition, their results also showed significant differences between BPA and BPS kinetics, with much higher systemic levels of active BPS than BPA, indicating that the replacement of BPA with BPS could lead to increased exposure to a hormonally active substance [133]. Furthermore, several *in vitro* studies showed the endocrine-disrupting potential of BPA-G and BPA-di-sulfate (BPA-DS) in GH3/B6/F10 rat pituitary cells [131] and the cytotoxicity of BPA-G in mice (3T3-L1) and human primary preadipocytes [129].

The main host enzymes involved in the human biotransformation of BPs to BPs-G are hepatic UGT12B15 and UGT1A9 [134,135]. UGT2B15 showed the highest activity in terms of BPA glucuronidation at low (1 µM) and high (20 µM) substrate concentrations [135]. Regardless, several extrahepatic UGTs including UGT1A7, UGT1A8, UGT1A10, UGT2A1, and UGT2A2 have shown a capacity to glucuronidate BPA, although mostly with lower activity than UGT2B15 [136]. In previous *in vivo* studies, BPA-G has been identified to be the predominant metabolite in rats, monkeys, and humans [134,137]. At one point, in rat liver microsomes, BPA was mainly glucuronidated by UGT isoform UGT2B1. Yokota et al. [138] indicate that glucuronidation plays a crucial role as a primary detoxification pathway closely linked to the metabolism and toxicity of BPA in mammals. Disruptions in the activity of hepatic UGT enzymes may lead to elevated concentrations of unconjugated BPs in the body (Figure 2). For example, reduced BPA glucuronidation activity has been linked to the UGT1A1*28 genotype, correlating with an increased risk of breast cancer in women [135,139,140]. Therefore, genetic polymorphisms and variability in the excretion of BPs-G/S forms in urine are critical factors to consider when biomonitoring bisphenol exposure and interpreting biomonitoring data.

Given the estrogenic potential and toxicity of both free and conjugated forms of BPs, in recent years, quantitative methods for measuring conjugated metabolites of BPA and BPS in human urine and blood were developed [128], and their concentration was evaluated in general populations, including in human maternal and cord serum [69].

### 4.2. Gut Microbial Metabolism

Recent evidence clearly demonstrates that the GM takes part in the metabolism of a wide range of ingested xenobiotics (i.e., drugs, heavy metals, and chemicals) into metabolites with altered activities [124]. The metabolism of xenobiotics by the GM is proposed as phase IV [141], as chemically modified compounds in phases I and II of host metabolism are further transformed in the large intestine via a broad range of microbial enzymes. The direct biotransformation of ingested xenobiotics occurs when these compounds reach the intestinal lumen, where they are acted upon by various bacterial enzymes capable of conducting diverse chemical reactions. The enzymatic repertoire represented by microbiotic enzymes in the human intestine is complex and includes various types of enzymes such as beta-glucosidases, beta-glucuronidases, aryl sulfatases, azoreductases, and nitroreductases. This diverse enzymatic arsenal contributes to the transformation of xenobiotics through a comprehensive set of chemical reactions.

**Figure 3:**
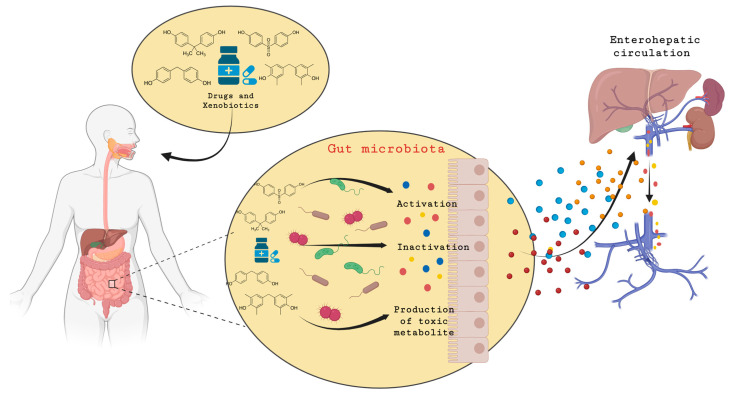
Gut microbial metabolism. Microorganisms inhabiting the human intestines alter the chemical structure of ingested compounds, including dietary components, industrial chemicals, and drugs. These changes affect the toxicity, biological activity, and bioavailability of xenobiotics. The mechanisms of the gut microbial manipulation of xenobiotic metabolism include (1) the inactivation of active xenobiotic metabolites by the gut microbiota, (2) the bioactivation of xenobiotic precursors by the gut microbiota, and (3) the reactivation of the detoxified host metabolites of xenobiotics that have reentered the colon through enterohepatic circulation.

The GM metabolizes xenobiotics differently than the host, primarily through reducing and hydrolytic enzymes, influencing their toxicity, lifespan, and bioavailability [142]. Host enzymes mainly perform oxidation and conjugation processes, while the enzymatic reactions of the microbiota are primarily reduction and hydrolysis [143,144]. Hydrolases, such as glycosidases, proteases, and sulfatases, catalyze the breakdown of complex compounds by adding a water molecule to the substrate, leading to bond cleavage. For instance, glycosidases break glycosidic bonds, releasing sugars [120,145]. Demethylation is another example of the disparities between host and microbiome xenobiotic metabolism, where host demethylation increases the polarity of a compound for excretion, while for microorganisms, it serves as a carbon source for growth. The biotransformation of xenobiotics by the GM influences their bioavailability, toxicity, and biological activity. The GM modifies the metabolites of xenobiotics produced by the liver, potentially restoring the original xenobiotic or creating new toxic metabolites. This occurs when nonpolar, lower-molecular-weight molecules are formed, making them easily reabsorbed. This process, known as ”hepato-intestinal circulation,” involves the reabsorption of these molecules and their return to the liver, controlling substrate storage and reuse in the body (Figure 3). Additionally, it may elevate the toxicity of some chemicals due to their extended half-life in the human body. Examples include drugs like diclofenac and irinotecan, where bacterial microbiota can generate toxic forms, leading to side effects such as diarrhea [123].

In turn, *in vivo* trials showed that LAB stimulated intestinal peristalsis, facilitating the elimination of chemicals from the body with feces [146]. Oishi et al. [146] showed that *Bifidobacterium breve* and *Lactobacillus casei* can reduce the intestinal absorption of BPA in rats. After the oral administration of probiotics, the concentration of BPA in the blood was significantly reduced and the amount of BPA eliminated with feces was significantly increased.

Additionally, the main functions of probiotic strains include the production of beneficial metabolites, such as SCFAs, which reduce the pro-inflammatory activity of the immune system, produce beneficial proteins/peptides, and reduce pathogenic toxins [83]. Probiotic strains can also strengthen the function of the intestinal barrier and regulate the tight junctions of the small intestine, making it difficult for chemical contaminants to penetrate the body. For example, an administration of *Lactobacillus plantarum* reversed the reduced mRNA expression of tight junction proteins (ZO-1, ZO-2, occludin, and claudin-1) caused by exposure to Cd, thus reducing intestinal permeability and the migration of Cd into systemic circulation [147].

The use of live cells (probiotics) and bioactive metabolites (postbiotics) presents a promising strategy for the food industry. Biological detoxification offers several advantages, including low cost, a broad range of target compounds, and minimal impact on nutrient components. The capacity of probiotics to efficiently detoxify BPA and other chemical pollutants could lead to the development of BPA removal systems, which could be safely incorporated as additives in food or feed for both animals and humans [148].

### 4.3. Bioadsorption

Physical adsorption is a reversible, metabolically passive physicochemical process, so chemicals can interact with both living and dead microbial cells [20,148]. LAB, due to the presence of polysaccharides and peptidoglycans in the cell wall, can adsorb these cells [149,150]. Wang et al. (2021) noted increased LAB binding activity with strains that have a larger specific surface area and cell wall volume [66]. Zhu et al. [151] showed enhanced BPA binding after treating LAB strains with hydrochloric acid and heat. Heat-killed cells, especially *Lactobacillus acidophilus* and *L. plantarum*, exhibited the highest BPA binding (70.25% and 70.26%, respectively) [151]. Oishi et al. [146] found a similar trend, suggesting that BPA detoxification is due to hydrophobic binding by LAB strains rather than enzymatic mechanisms.

Moreover, the biosorption of xenobiotics to the cell wall is strain-specific. For example, *Lactobacillus* and *Bifidobacterium* have the greatest potential for binding chemical pollutants [149]. Shoukat et al. [152] confirmed in their research that LAB, such as *Bifidobacterium*, can bind to carcinogenic substances like benzo[a]pyrene. They demonstrated this through adsorption, in which the peptidoglycan contained in the bacterial cell wall formed a strong covalent bond with the benzo[a]pyrene found in food, thus removing it. Similarly, Zhu et al. [151] conducted a study on the ability of six strains of LAB isolated from dairy fermentation to remove bisphenol A from phosphate-buffered saline. Among these strains, *L. acidophilus* and *L. plantarum* were more effective than others, reducing the content of bisphenol A by 48.44% and 50.80%, respectively, after 24 h of incubation [151] (Table 1).

The absorption of xenobiotics into the LAB cell wall is facilitated by the negative charge on their surface resulting from various negatively charged functional groups like carboxyl or phosphoryl groups [20]. In a study by Endo et al. [153], nine *Lactococcus* strains were examined for their ability to remove BPA and its analogs from growth media. The tested BPA analogs included 4,4′-biphenol (BP), bisphenol A diacetate (BPAA), bisphenol A dimethyl ether (BPAM), 4,4′-dihydroxydiphenylmethane (DDM), 4,4′-ethylidenebisphenol (EBP), and 2,2′-diphenylpropane (DPP). *Lactococcus lactis* subsp. *lactis* strain 712 displayed the highest efficacy in removing BPA through adsorption. Its efficiency varied depending on the type of BPA analog, with the absence of a dimethyl group negatively impacting removal (1.4% and 2.2% for DDM and EBP, respectively), compared to 3.8% for BPA. The acetylation of the OH group in BPAA and its replacement with a methyl ether group in BPAM resulted in a lower removal efficiency (4.4% and 9.2%, respectively) than BPA. Conversely, DPP, lacking an OH group, exhibited the highest removal efficiency at 38%. These findings suggest that the presence of an OH group in BPA may hinder its adsorption by *Lactococcus*, while hydrophobic substitutes may enhance it [153] (Table 1). Solouki et al. [154] conducted research on the effectiveness of multispecies probiotic supplements in BPA bioremediation, observing an 80% decrease in BPA concentration within the first hour, with the highest efficacy seen in the synergy of *L. acidophilus* and *L. plantarum* strains (Table 1).

### 4.4. Biodegradation

The biodegradation process, crucial for pollution removal, relies on the role of bacteria and fungi with enzymatic degradation capabilities. In a study by Kyrila et al. [157], it was found that the biodegradation process depends on bacterial metabolism and yields substances such as hydroquinone (HQ), 4-hydroxyacetophenone (HAP), 4-hydroxybenzoic acid (HBA), and 4-isopropenylphenol (PP). HBA was present in all microorganisms tested, except *Bacillus subtilis*, while PP was exclusively found in *B. subtilis*. The accumulation of HQ in all strains suggests a slower degradation process or transformation [155,157]. A similar phenomenon was observed in BPA’s degradation by *Achromobacter xylosoxidans*, possibly due to variations in enzyme activity [155]. However, there is a lack of data on the toxicity of these metabolites. It has been shown that addition products are detectable intracellularly in small amounts. Their low levels may result from their further biodegradation into simpler molecules, mainly HQ, HAP, and HBA, subsequently undergoing metabolism for mineralization [157] (Table 1). Notably, one of the metabolites is isopropenylphenol, which is transformed into p-hydroquinone and then mineralized by both fungi and bacteria [155]. Monomers resulting from BPA biodegradation exhibit low toxicity compared to BPA and are non-mutagenic [51].

In another study, the ability of six commonly used probiotics, namely Limosilactobacillus reuteri (formerly Lactobacillus reuteri), Lactobacillus helveticus, Levilactobacillus brevis (formerly Lactobacillus brevis), Lactobacillus delbrueckii, Lacticaseibacillus casei (formerly Lactobacillus casei), and B. subtilis, to degrade BPA in food packaging was assessed. L. reuteri exhibited the highest degradation level, reaching approximately 69.83%. The study also investigated the impact of this strain on reducing BPA levels in beverages such as black tea, orange juice, and cold mung bean tea stored in cans. The BPA levels decreased by 92.74%, 92.33%, and 92.33% after one day of storage, respectively, and reached 0% after 27 days. Moghaddam et al. [156] demonstrated that the probiotic bacteria *L. plantarum* and *L. acidophilus* effectively reduced BPA levels in yogurt during storage (Table 1). The concentrations of BPA in the yogurt were analyzed over a 4-week storage period, revealing that *L. plantarum* and *L. acidophilus* reduced BPA levels in yogurt by 82.80% and 43.44%, respectively. At the end of the storage period, the reduction efficiency was higher for *L. plantarum*, reaching as high as 95.30%. Biological degradation offers advantages such as a low cost, a broad range of target compounds, and minimal side effects for nutrient components [148].

## 5. Next-Generation Probiotics (NGPs) as a New Tool

Traditional probiotics like Bifidobacterium and Lactobacillus, although biologically safe, have shown limited efficacy in alleviating certain diseases, particularly obesity and metabolic disorders. Generally, traditional probiotics have been chosen randomly or based on widely available research and practical experience. Unfortunately, their overall impact and functions in disease mitigation are statistically marginal. Despite their widespread use, there is a lack of conclusive evidence of their efficacy, leading to an urgent need to identify specific NGPs for particular conditions [25,39,158]. In recent years, advancements in research and the utilization of next-generation sequencing platforms and bioinformatics tools have facilitated the intensive exploration of potential NGPs. Among the candidates are *Faecalibacterium prausnitzii*, *Akkermansia muciniphila*, and *Parabacteroides goldsteinii* [159,160]. Unconventional newly identified GM bacteria for therapeutic purposes are gaining more attention compared to traditional probiotics. However, several challenges must be addressed before NGPs can be widely implemented in clinical practice. These NGPs can be considered drugs (live biotherapeutic products, LBPs), which require much more rigorous classifications [25]. Further research on NGPs is essential, including evaluating their effectiveness in treating target diseases; physiological, genomic, and metabolomic properties; and potential virulence factors and host interactions [25]. Additionally, understanding the genetic characteristics of the bacteria and their physiological traits, including their growth dynamics and antibiotic sensitivity patterns, is necessary. Before applying NGPs, a series of clinical trials must be conducted to determine their safety, dosage ranges, side effects, and benefits. Most potential NGP candidates also face issues related to nutritional requirements and their sensitivity to oxygen conditions, which pose technological challenges to their large-scale production. Therefore, developing appropriate delivery systems to enhance the viability and functionality of probiotic strains is necessary [161,162].

Currently, there is a lack of studies on the neutralization of dietary obesogens by NGPs and their impact on health. Nonetheless, they have significant prospects in this area, given that identified next-generation microorganisms such as *F. prausnitzii*, *A. muciniphila*, and *P. goldsteinii* show a negative correlation with obesity, diabetes, and low-grade inflammation [160,163]. NGPs offer significant potential in the treatment of metabolic disorders through the modulation of the GM. As previously mentioned, a healthy digestive system acts as a selective barrier, allowing the absorption of nutrients while blocking gut bacteria and xenobiotics from entering the bloodstream. Exposure to bisphenols causes GM dysbiosis, compromising the integrity of the gut barrier, reducing the expression of tight junction proteins, and increasing gut permeability, endotoxin levels, and pro-inflammatory cytokine secretion. Recent studies on NGPs show that they significantly reduce pro-inflammatory cytokine levels, increase the expression of tight junction proteins, and improve the metabolic profile and mucus layer thickness, thus supporting the epithelial barrier by regulating anti-inflammatory pathways [24]. This suggests that NGPs may have the potential to prevent inflammatory conditions and treat obesity and other metabolic diseases resulting from chronic exposure to dietary obesogens.

### 5.1. Faecalibacterium prausnitzii

*Faecalibacterium prausnitzii* is a Gram-positive bacterium belonging to the *Ruminococcaceae* family and the *Firmicutes* type. It is a consumer of acetate, ferments glucose, and produces SCFAs such as butyrate, propionate, and D-lactate [25]. It is one of the most common intestinal bacteria (more than 5%) and contributes to the interaction between the microbiota and the digestive tract. Various studies have linked *F. prausnitzii* to overall health status. A decrease in the presence and abundance of *F. prausnitzii* has been associated with intestinal disorders such as inflammatory bowel disease, irritable bowel syndrome, colorectal cancer, and obesity [164].

*Faecalibacterium* demonstrates anti-inflammatory properties, which have been well documented in diseases such as Crohn’s disease (CD) [163] and multiple sclerosis [165]. The anti-inflammatory effects of *F. prausnitzii* are attributed to its release of metabolites, including butyrate [166,167]. These metabolites can inhibit the activation of nuclear factor kappa B (NF-kB), which is involved in the transcription of *F. prausnitzii* genes, increasing its presence in the gut mucosa of CD patients and supporting the production of anti-inflammatory cytokine IL-8 [168]. Additionally, the anti-inflammatory profile of *F. prausnitzii* is characterized by its ability to produce very low levels of IL-12 and secrete IFN-γ, while simultaneously promoting high levels of IL-10. Moreover, the IL-10/IL-12 ratio is often used to determine whether a strain exhibits anti-inflammatory or pro-inflammatory properties. *F. prausnitzii* displays an anti-inflammatory profile, with a higher IL-10/IL-12 ratio compared to other probiotic strains [168].

Furthermore, due to its butyrate production, *F. prausnitzii* effectively mitigates the inflammation caused by an exposure to EDCs. Butyrate enhances the functions of the intestinal epithelial barrier and the integrity of tight junctions between epithelial cells, preventing the translocation of toxins, pathogens, and antigens into the gut’s circulation. As a result, this limits gut permeability and protects against the development of chronic inflammatory states [169,170,171]. Moreover, studies have confirmed the anti-inflammatory effects of *F. prausnitzii* in postbiotic form, demonstrating its protective role in inflammation by significantly reducing the production of pro-inflammatory cytokines such as IL-12 while increasing anti-inflammatory cytokines like IL-10 [168]. The ability of *F. prausnitzii* to prevent acute colitis in mice has also been studied. Severe colitis was observed in a control group with induced colitis. The daily oral administration of live *F. prausnitzii* (5 × 10^9^ CFU) significantly improved colonic inflammation, reducing weight loss [168].

Increased inflammation caused by reduced epithelial barrier integrity in the gut may result from the dysbiosis of the GM. This condition can be triggered by the consumption of processed foods containing obesogens that affect the GM, promoting adipogenesis, weight gain, and microbiome dysbiosis. Research suggests that *F. prausnitzii* plays a crucial role in maintaining gut homeostasis. Both live *F. prausnitzii* and postbiotics derived from it have been shown to alleviate dysbiosis [168]. In dysbiosis, the balance between beneficial and pathogenic bacteria is disrupted, leading to the overgrowth of pathogenic bacteria such as *Escherichia coli* or *Clostridium*. *F. prausnitzii* restores microbiota balance by limiting the growth of pathogenic bacteria. The production of butyrate and other SCFAs by *F. prausnitzii* inhibits pathogen development, creating an unfavorable environment for their proliferation while promoting the restoration of diversity and supporting a healthy gut environment that fosters the growth of other beneficial bacteria [172]. Additionally, *F. prausnitzii* works synergistically with other beneficial gut bacteria, such as *A. muciniphila*. By strengthening mutual interactions between these microorganisms, it supports gut health, leading to more effective counteraction against dysbiosis and inflammation [173].

Research highlights the important role of *F. prausnitzii* in regulating metabolism, particularly in the context of obesity and metabolic health. Recent studies suggest that the amount of *F. prausnitzii* in feces is lower in obese mice but can be increased using anti-obesity agents [174]. In a study conducted by Yang et al. [102] on a high-fat diet (HFD)-induced obesity mouse model, strains of *F. prausnitzii* significantly reduced weight gain, liver and fat mass, and caloric intake [102]. They also improved lipid and glucose metabolism in the liver and adipose tissue, confirmed by the regulation of gene expressions related to lipid metabolism, such as ACC1, FAS, SREBP1c, leptin, and adiponectin. Mice on a high-fat diet treated with *F. prausnitzii* showed reduced fat accumulation in the liver and a smaller adipocyte size compared to the control group. Additionally, lipid measurements, liver histology samples, and AST and ALT levels in *F. prausnitzii*-treated and control mice were correlated, suggesting that these mice had healthier livers than the control group on a high-fat diet [168].

Further research is needed on *F. prausnitzii* to better understand its action and determine the best ways to use it in humans, including its dose and forms of administration. Further clinical trials in humans are necessary to validate the safety and effectiveness of *F. prausnitzii* and to confirm the beneficial effects observed in animal models and research on other target species.

### 5.2. Akkermansia muciniphila

*Akkermansia muciniphila*, belonging to the *Verrucomicrobia* phylum, is another key candidate for probiotics. It is a common bacterial component of the human digestive tract, comprising up to 5% of the total bacterial microbiome and making up more than 1% of all fecal cells [160,175]. The mucus layer that covers the intestines provides many benefits for intestinal bacteria. It acts as a direct nutrient source for these bacteria, particularly in the large intestine, where alternative carbon sources are limited. *A. muciniphila* is a bacterium that breaks down mucin, which is found in the mucus layer and serves as a protective barrier against xenobiotics in the intestine. It was first isolated in 2004 from a fecal sample of a healthy person, with mucin serving as the only source of carbon, nitrogen, and energy. [175].

Research findings show that the levels of *A. muciniphila* are strongly correlated with lipid metabolism markers and inversely associated with conditions such as obesity, diabetes, and inflammatory bowel diseases, which may result from exposure to EDCs. These conditions are linked to impaired gut barrier function, leading to increased LPS levels in the blood and ultimately causing inflammatory and metabolic disorders [163,174]. Schneeberger et al. [176] confirmed the purported beneficial impact of *A. muciniphila* on metabolism. They found that in mice fed a lard-enriched diet, the levels of *A. muciniphila* significantly decreased, while a fish oil-enriched diet substantially increased its presence in the gut. This effect was associated with improved gut barrier function and reduced fat mass. *A. muciniphila* has a beneficial impact on energy metabolism, making it an important factor in obesity prevention.

Obesity is one of the key health issues related to EDC exposure. Human studies have provided evidence of a negative correlation between *A. muciniphila* levels and overweight, obesity, or hypertension. It has been shown that daily oral supplementation of 10^10^ live or pasteurized *A. muciniphila* bacteria for three months was safe and well tolerated. Interestingly, supplementation with pasteurized *A. muciniphila* resulted in slight weight loss (−2.27 ± 0.92 kg) compared to the placebo group, along with a reduction in fat mass (−1.37 ± 0.82 kg) and hip circumference (−2.63 ± 1.14 cm) compared to baseline values. After three months of supplementation, markers of liver dysfunction and inflammation in the blood decreased, while the GM’s structure remained unchanged. The study demonstrated that supplementation with *A. muciniphila* was both safe and well tolerated [177].

Similarly, Everard et al. [178] showed that *A. muciniphila* levels decreased in obese mice and mice with type 2 diabetes. Moreover, studies demonstrated that administering *A. muciniphila* could reverse high-fat-diet-induced metabolic disturbances, such as fat mass gain, metabolic endotoxemia, and adipose tissue inflammation, through the secretion of endocannabinoids. These endocannabinoids regulate inflammation, maintain gut barrier integrity, and influence the secretion of gut peptides [178].

*A. muciniphila* plays a key role in alleviating gut barrier dysfunction, which is a major cause of various metabolic issues, such as inflammation. This process occurs by mitigating gut barrier damage through the inhibition of pro-inflammatory cytokines (including TNF-α, IL-1β, and IL-6) and the production of endotoxins/LPS [68]. All of these mechanisms correlate with more efficient energy utilization, as evidenced by reduced inflammation and improved insulin resistance [177,178]. It has also been shown that both live and pasteurized bacteria, as well as the specific Amuc 1100 protein isolated from the outer membrane of *A. muciniphila*, which interacts with the Toll-like receptor 2 (TLR2), improve gut barrier function and restore the proper expression of tight junction proteins. This is associated with a thicker mucous layer and improved metabolic disorders [164,178,179].

*A. muciniphila* plays a crucial role in eliminating intestinal barrier dysfunction, which is the primary cause of diverse metabolic issues such as inflammation, fat accumulation, and adipose tissue inflammation. It does so by mitigating damage to the intestinal barrier through the inhibition of pro-inflammatory cytokines (including TNF-α, IL-1β, and IL-6) and the production of endotoxins/LPS [68].

The anti-inflammatory effects of an oral administration of live and pasteurized *A. muciniphila* (1 × 10^8^ CFU) were confirmed by studies conducted by Hu et al. [179], where this supplementation significantly lowered IL-6 and IL-1β levels and increased the expression of IFN-γ, IFN-β, and IL-10 in mice infected with the H7N9 influenza virus. This suggests that the anti-influenza role of *A. muciniphila* is due to its anti-inflammatory properties [180]. Naisiri et al. [180] also confirmed that both live and inactivated *A. muciniphila* can alleviate the cytotoxic effects of *C. difficile*, modulate intestinal inflammation, and relatively improve gut barrier dysfunction in Caco-2 cells *in vitro*. The strong anti-inflammatory properties of *A. muciniphila* were confirmed in studies of sepsis patients. Supplementation with live strains and culture supernatant reduced sepsis-induced mortality. It was shown that live bacteria could generate a new tripeptide, Arg-Lys-His (RKH), which reduces inflammatory cell activation [181].

In 2015, at the request of the European Commission’s EFSA on Nutrition, in accordance with Regulation (EU) 2015/2283 on new foods and food allergens, an opinion was issued on pasteurized *A. muciniphila* as a new food. *A. muciniphila* has been proposed for use as a dietary supplement at a maximum dose of 5 × 10^10^ cells per day for adults, excluding pregnant and breastfeeding women. The panel found that the production process of the new food was well described and the information provided about its composition was sufficient. Considering the composition of the new food and the proposed conditions of its use, its consumption is not harmful to health. Based on available data and assuming a uncertainty factor of 200, the panel concluded that an intake of 3.4 × 10^10^ cells per day is safe for the target group of consumers provided that the number of viable cells in the new food is less than 10 CFUs per gram (EFSA Panel on Nutrition, Novel Foods and Food Allergens (NDA) 2021).

Studies on *A. muciniphila* highlight its potential in treating diabetes, obesity, and metabolic diseases. However, due to its strictly anaerobic nature and sensitivity to oxygen, challenges arise in industrial-scale production. Understanding how the diverse functions of *A. muciniphila*, along with the complexity of the human GM, impact health is crucial, as is maintaining its balance. To better comprehend and harness the potential of *A. muciniphila*, there is a need for intensified clinical and experimental research providing comprehensive data on the safety and effectiveness of its application.

## 6. Conclusions

Diet plays a significant role in body weight regulation, and NGPs are a key component of the body’s metabolic response. Daily exposure to low doses of various chemicals can lead to “cocktail effects”, making it challenging to assess their impact on health. Many new chemicals introduced into the industry have not been thoroughly tested for their potential adverse effects.

Chronic exposure to xenobiotics disrupts the GM, affecting the host’s metabolic pathways and leading to diseases such as obesity. Preventive and therapeutic measures are necessary to restore disrupted homeostasis by addressing the interactions between xenobiotics and the GM.

NGPs, such as *Akkermansia muciniphila* and *Faecalibacterium prausnitzii*, are being intensively studied due to their potential health benefits, which may surpass those offered by traditional bacterial strains. They play a crucial role in restoring and maintaining homeostasis—the key biological balance within the body. Their role in neutralizing obesogens is becoming increasingly clear. NGPs can act directly by binding to these substances and converting them into less harmful forms and indirectly by reducing inflammation and strengthening the gut barrier, which protects against toxins entering the bloodstream. By restoring homeostasis, NGPs counteract the negative effects of EDCs, contributing to improved immune, gut, and metabolic function. Maintaining this balance is essential for health and protects the body from the long-term effects of environmental chemicals.

NGPs may play a pivotal role in personalized medicine, allowing for the development of individualized probiotic therapies. However, before they can be widely implemented, extensive clinical trials are needed to confirm their efficacy and safety, particularly in individuals with chronic diseases. Bringing these probiotics to the market also requires appropriate regulatory measures. Despite their research challenges, NGPs offer new opportunities for combating obesity and its related complications.

## Figures and Tables

**Table 1:** Efficiency of bacterial strains in removing BPA and its analogs through bioadsorption and biodegradation.

Reference	Compound	Matrix	Incubation Conditions, Removal Mechanism	Microorganism	Concentration of Compound	Removal Ratio [%]
Endo [153] (2007)	DPP	Medium	1 h at 30 °C, Bioadsorption	*L. lactis* subsp. *lactis 712*	2 (μg/mL)	38.4 ± 1.1
	BP					1.2 ± 0.2
	DDM					1.3 ± 0.2
	EBP					2.2 ± 0.8
	BPA			*L. lactis* subsp. *cremoris C60*		17.2 ± 1.6
				*L. lactis* subsp. *Lactis 527*		18.9 ± 0.4
				*L. lactis* subsp. *lactis 712*		22.4 ± 6.5
				*L. lactis* subsp. *lactis 712*		3.8 ± 1.0
				*L. lactis* subsp. *lactis bv. diacetylactis 8W*		13.1 ± 1.8
				*L. lactis* subsp. *lactis bv. diacetylactis C66*		16.5 ± 1.2
				*L. lactis* subsp. *lactis bv. diacetylactis DRC1*		9.1 ± 8.1
				*L. lactis* subsp. *lactis bv. diacetylactis H59*		11.1 ± 3.0
				*L. lactis* subsp. *lactis bv. diacetylactis N7*		18.0 ± 0.5
				*L. lactis* subsp. *lactis G46*		11.7 ± 1.8
				*L. lactis* subsp. *lactis H46*		9.9 ± 4.1
	BPAA			*L. lactis* subsp. *lactis 712*		4.4 ± 1.5
	BPAM					9.2 ± 0.3
Zhu [151] (2017)	BPA	PBS solution	Acid-treated for 1.5 h, Bioadsorption	*L. acidophilus*	5 (mg/L)	66.33 ± 0.20
				*L. bulgaricus*		47.12 ± 1.02
				*L. paracasei*		62.45 ± 0.48
				*L. plantarum*		61.84 ± 0.41
				*L. rhamnosus*		45.94 ± 0.13
				*S. thermophilus*		35.77 ± 0.70
			Heat-treated for 24 h at 120 °C, Bioadsorption	*L. acidophilus*		70.25 ± 0.75
				*L. bulgaricus*		54.78 ± 0.65
				*L. paracasei*		67.89 ± 0.64
				*L. plantarum*		72.26 ± 0.36
				*L. rhamnosus*		51.11 ± 0.51
				*S. thermophilus*		37.87 ± 0.67
			Viable for 24 h at 30 °C, Bioadsorption	*L. acidophilus*		48.44 ± 0.36
				*L. bulgaricus*		33.17 ± 0.57
				*L. paracasei*		40.28 ± 0.56
				*L. plantarum*		50.80 ± 0.24
				*L. rhamnosus*		27.94 ± 0.29
				*S. thermophilus*		24.48 ± 0.80
Solouki [154] (2018)	BPA	Saline basal medium	24 h at 37 °C, Bioadsorption	Familact, *L. casei*, *L. acidophilus*, *L. rhamnosus*, *L. bulgaricus*, *B. breve*, *B. longum*, *S. thermophilus*	0.5 (mg/L)	86.06 ± 0.55
				Gerilact, *L. casei*, *L. acidophilus*, *L. rhamnosus*, *L. bulgaricus*, *B. breve*, *B. longum*, *S. thermophilus*		87.70 ± 0.49
				Kidilact zink, *L. casei*, *L. acidophilus*, *L. rhamnosus*, *L. bulgaricus*, *B. breve*, *B.infantis*, *S. thermophilus*		24.96 ± 0.10
				Kidilact, *L. casei*, *L. acidophilus*, *L. rhamnosus*, *L. bulgaricus*, *B. breve*, *B.infantis*, *S. thermophilus*		85.67 ± 0.44
				Lactocare, *L. casei*, *L. acidophilus*, *L. rhamnosus*, *L. bulgaricus*, *B. breve*, *B. longum*, *S. thermophilus*		92.00 ± 0.82
				Lactofem, *L. acidophilus*, *L. plantarum*, *L. fermentum*, *L.gasseri*		88.11 ± 0.47
Ju [155] (2019)	BPA	Black tea beverage	48 h at 37 °C, Biodegradation	*L. reuteri*	31.7 μg/L	92.74
		Orange juice beverage			31.3 μg/L	92.33
		Mung bean cold tea			31.4 μg/L	92.33
Moghaddam [156] (2020)	BPA	Yogurt	28 d at 37 °C, Biodegradation	*L. acidophilus*	54.36 mg/L	90.77
				*L. plantarum*	36.64 mg/L	95.30
Kyrila [157] (2021)	BPA	Medium	96 h at 30 °C, Biodegradation	*B. subtilis*	23.78 ± 0.29 (μg/mL)	51.90
				*E. faecalis*	27.16 ± 0.21 (μg/mL)	45.30
				*L. lactis*	29.54 ± 0.15 (μg/mL)	39.10
				*L. plantarum*	28.15 ± 0.59 (μg/mL)	41.60
				*S. cerevisiae*	27.51 ± 0.17 (μg/mL)	44.20
